# The Impact of the COVID-19 Pandemic and Socioeconomic Deprivation on Admissions to the Emergency Department for Psychiatric Illness: An Observational Study in a Province of Southern Italy

**DOI:** 10.3390/life13040943

**Published:** 2023-04-03

**Authors:** Massimo Giotta, Francesco Addabbo, Antonia Mincuzzi, Nicola Bartolomeo

**Affiliations:** 1 School of Medical Statistics and Biometry, Department of Interdisciplinary Medicine, University of Bari Aldo Moro, 70124 Bari, Italy; 2 School of Medical Statistics and Biometry, University of Bari Aldo Moro, Azienda Sanitaria Locale Taranto, 74121 Taranto, Italy; francescodr.addabbo@gmail.com; 3 Unit of Statistics and Epidemiology, Azienda Sanitaria Locale Taranto, 74121 Taranto, Italy; antonia.mincuzzi@asl.taranto.it; 4 Department of Interdisciplinary Medicine, University of Bari Aldo Moro, 70124 Bari, Italy; nicola.bartolomeo@uniba.it

**Keywords:** COVID-19, pandemic, psychiatric illness, emergency department, admission rate, public health restrictions, socioeconomic deprivation, deprivation index

## Abstract

The restriction measures adopted to limit population movement in order to contain the COVID-19 pandemic contributed to a global public health system crisis. This retrospective study aimed at identifying changes in psychiatric admissions to Accident and Emergency Departments (A&Es) in a province in southern Italy during the first two years of the pandemic and was characterized by two different restriction levels (phases 2 and 3) compared to the pre-pandemic period (phase 1). We also investigated the role of socioeconomic deprivation (DI) on psychiatric admissions. The total number of patients admitted to the A&Es was 291,310. The incidence of admission for a psychiatric disorder (IPd) was 4.9 per 1000 admissions, with a significant younger median age of 42 [IQR 33–56] compared to non-psychiatric patients (54 [35–73]). The type of admission and type of discharge were factors related to the psychiatric admission to A&E, and their relationship was modified by the pandemic. In the first year of the pandemic, patients with psychomotor agitation increased compared to the pre-pandemic period (72.5% vs. 62.3%). In the period preceding the spread of SARS-CoV-2, the IPd was equal to 3.33 ± 0.19; after the pandemic started, there was an increase in the IPd: 4.74 ± 0.32 for phase 2 and 3.68 ± 0.25 for phase 3. The IPd was higher for psychiatric admissions from areas with a very low DI compared to areas with a low DI; however, during phase 2, this difference was reduced. In conclusion, an increase in admissions for psychiatric disease was observed during the initial spread of SARS-CoV-2. Patients who lived in the most deprived municipalities generally came to the A&Es less than others, probably because the patients and their families had less awareness of their mental health. Therefore, public health policies to address these issues are needed to reduce the pandemic’s impact on these conditions.

## 1. Introduction

By 31 December 2021, 6.1 million cases of SARS-CoV-2 had been recorded in Italy, with 13,740 deaths reported since February 2020 [1]. Moreover, on the grounds that in February 2020, Italy was one of the countries with the very highest spread of SARS-CoV-2 instances [2,3], the government imposed a series of public health restrictions to mitigate the further spread of the infection. With the unprecedented and rapid spread, public healthcare capacities, which were already reduced due to the shortage of health professionals, shifted toward COVID-related emergencies. Furthermore, healthcare professionals had to cope daily with ethical decisions regarding non-discriminatory access to healthcare facilities, in a scenario with a shortage of personal protective equipment, the rationing of medical devices, and insufficient hospital beds in intensive care units [4,5]. The imposed restrictions applied during the first two years of the pandemic caused a change in people’s lifestyles, underlined by many studies, which highlighted the direct and indirect effects of the spread of SARS-CoV-2 [6,7,8]. In the first period, the lockdown restrictions and the fear of being infected determined a reduction in hospitalization for both chronic and acute diseases [9] and a significant increase in hospital deaths related [10,11] and unrelated to COVID-19 infection [12]. A significant reduction in hospital admissions was recorded for all diseases, including those that were time-dependent (e.g., coronary acute syndrome and stroke) and those requiring careful clinical and specialist monitoring [9]. Several concerns arose in Italy when it was observed that the number of patients admitted for psychiatric or psychological issues and the suicide rate increased together during the first phases of the total lockdown [13]. An increase in the suicide rate was observed in more states after the first spread of SARS-CoV-2. Prior to the pandemic, the prevalence of suicidal ideation across the world was approximately 2.0% [14]. However, a meta-analysis of international studies has revealed that during the pandemic, the pooled prevalence of suicidal ideation increased dramatically to 10.8% [15]. In Canada, the prevalence of suicidal ideation was 2.4% in the fall of 2020 [16]. Unfortunately, this number increased to 4.2% in the spring of 2021, which is significantly higher than the 2.7% prevalence of suicidal ideation reported in 2019 [17]. These findings underscore the significant impact that the pandemic has had on mental health, particularly with regard to suicidal ideation [18].This effect has been observed similarly in other pandemics [19]. For these reasons, some international groups of mental health experts [20] reflected on the challenges of mental health and COVID-19 and how this crisis could be shifted to an opportunity.

Furthermore, it is important to underline that all the pandemic periods were associated with economic instability, which especially affected low-income and middle-income patients [21]. Furthermore, several studies have underlined that inequality is responsible for an increase in the risk of contracting contagious diseases [22,23,24,25], and this increase is proportional to the social deprivation [26]. More studies also underline the relationship between socioeconomic deprivation and mental illness as suicide [27,28] and depression [29,30]. To evaluate the socioeconomic impairment, we used an index created by Caranci et al. [31], which evaluated the level of education, home ownership, population density, unemployment, and the prevalence of single-parent families.

The first aim was to identify how the pandemic caused by SARS-CoV-2 and the different levels of restriction on public health services adopted during the pandemic period modified the admission rate in an Accident and Emergency (A&E) for psychiatric illnesses. The secondary aim was to evaluate how socioeconomic deprivation modified the effect of pandemic public restriction on the psychiatric admission rates of A&E units.

## 2. Materials and Methods

We conducted a retrospective observational study using anonymous aggregated data extracted from the administrative healthcare databases of the Local Health Authority (LHA) in Taranto province. The data were collected and stored in the Regional Information System. Access to data is regulated by a regional policy to allow the use of data for epidemiological purposes by the Taranto LHA’s Unit of Epidemiology and Statistics. Data were provided after anonymization and were treated according to the current national laws for the treatment of health data. We analyzed the registry of admission to the Accident and Emergency Departments (A&E) of the province of Taranto from 1 January 2019 to 31 December 2021. In this province, there are four A&Es, three sites in territorial hospitals and one site in a general hospital. The inclusion criteria were an age above 13 years, residence in a municipality of the province of Taranto, and every type of access to A&E.

An admission was codified as psychiatric only when the A&E triage nurses codified the principal issues as “psychiatric disorder” (regional A&E code 28), “self-harm” (regional A&E code 31), or “psychomotor agitation” (regional A&E code 16). In addition to the type of admission to A&E, the additional collected information was sex, age, type of access, and nationality and type of discharge.

Periodically, on the basis of 20 indicators (the most important are the Rt and the occupancy rates of hospital beds in intensive care), a national technical-scientific committee suggested the different levels of lockdown to the government. Therefore, the three-year period under analysis was divided into three macro phases with different levels of public health restrictions:

The three-year period under analysis was divided into three phases with different levels of public health restrictions:

1. Phase 1, from 1 January 2019 to 28 February 2020, the pre-pandemic period, without restrictions.
2. Phase 2, from 1 March 2020 to 31 December 2020, the first pandemic period, with a high level of restrictions: restrictions on leaving the house unless for essential purposes, cessation of educational services, and shutdown of all commercial establishments and public offices [32,33,34].
3. Phase 3, from 1 January 2021 to 31 December 2021, the second pandemic period, with a medium–low level of restrictions: suspension of educational indoor service, reduction of the number of people accessing commercial activities [35,36].

Psychiatric admissions to A&E versus other A&E admissions and psychiatric admissions to A&E between the three phases were compared. Categorical data were shown as frequency and percentage and the chi-square test or Fisher’s exact test was used, as appropriate, for comparison between groups. Quantitative data were expressed as means and standard deviation if normal distribution was verified, or median and interquartile range (IQR) if the assumption of normality was not acceptable. The Kolmogorov–Smirnov test was used to test normality. Student’s *t*-test or Mann –Whitney U test, as appropriate, was used for the comparison between independent groups. Pairwise comparisons between phases were adjusted by the Conover nonparametric method for quantitative variables and by Tukey method for categorical variables.

The crude rate of psychiatric admissions to A&E for each phase was calculated as the ratio between the number of psychiatric admissions to A&E and the total number of admissions to A&E multiplied by 1000.

The socioeconomic inequality was evaluated by the Deprivation Index (DI), updated in 2011 from Rosano et al. [37], calculated at the census level, based on 2011 census data. This index measured a combination of relative social disadvantages in the resident population: poor education, job shortage, poor housing and family conditions. The five indicators (X1–X5) of DI were calculated as follows:

X1 = (population with an education equal to elementary school, literate or illiterate)/(population aged 6 and over) × 100;

X2 = (unemployed or seeking their first job)/(workforce) × 100;

X3 = (homes occupied by renters)/(homes occupied by resident persons) × 100;

X4 = (total population)/(surface (m^2^) of dwellings occupied by resident persons) × 100;

X5 = (single father or mother with children, in single nuclear families, with and without isolated members)/(total families) × 100.

The DI is $DI=\overset{5}{\underset{i=1}{\sum }}X_{i}$ with $z_{i}=\frac{x_{i}-m_{x_{i}}}{S_{x_{i}}}$, where $m_{x}$ and $S_{x}$ are the average and the standard deviation of the indicator *x*. We calculated the municipal DI as a weighted average of the DIs of the census sections, using the resident populations in each census section as weights. The correlation between the municipality psychiatric admissions rate in A&E and municipal DI was calculated for each phase. The Mardia test was used to verify multivariate normality between municipal rates and DI, and consequently the nonparametric Spearman’s correlation coefficient was used.

The DI was then grouped and stratified in classes based on the quartiles of the distribution: high deprivation (H), DI less than −0.596; medium (M), DI between −0.596 and −0.194; low (L), DI between −0.194 and 0.341; and very low (VL), DI greater than 0.341.

A multivariable generalized linear model, as defined by Nelder and Wedderburn [38], using Poisson distribution with a logarithmic link function, was used to investigate the possible effects of the pandemic in association with the socioeconomic inequalities on the variation in the numbers of psychiatric admissions to A&E compared to the total number of admissions to A&E.

The covariates of the model were sex, age (divided into four groups: 13–20 years, 21–40 years, 41–60 years, and >60 years), the class of the DI, the phases of the pandemic, and the interaction between the class of the deprivation index and the phases of the pandemic. The results were shown as the rates and 95% confidence intervals (CI) for each level of each covariate using the inverse link function, averaging the levels of the other covariates. We also calculated the rate ratios with their 95% confidence intervals, which showed all pairwise variations in covariate values. The Tukey correction was used to adjust all pairwise multiple comparisons. A *p*-value less than 0.05 was considered statistically significant.

Data management, descriptive statistics and regression models were conducted using SAS/STAT version 9.4 for PC (SAS Institute, Cary, NC, USA).

## 3. Results

The total number of patients admitted to the A&Es in the province of Taranto was 369,967: 156,148 in 2019, 107,938 in 2020, and 105,611 in 2021. In total, 78,085 patients were excluded because they did not meet the inclusion criteria. The rate of admission for psychiatric disorders was 4.9 per 1000 admissions to A&E. The other admissions to A&E were predominantly for abdominal pain, chest pain, dyspnoea, heart disease, etc. The main characteristics of the patients under analysis are shown in Table 1.

The median age was 54 years (IQR 35–73) for all patients admitted to A&E. The psychiatric patients were younger (42 years vs. 54, *p* < 0.001) and predominantly male (62.2% vs. 48.7, *p* < 0.001) compared to the non-psychiatric patients, while no difference was found for nationality. The admission to A&E was mainly by ambulance after calling the emergency telephone number (118) for the psychiatric patients; in contrast, the others arrived at the hospital on their own (*p* < 0.001). The distribution of admission in the DI classes was different between the psychiatric patients and the other patients (*p* < 0.001), with the psychiatric patients admitted to A&E coming primarily from municipalities with very low socioeconomic deprivation (60.5% vs. 40.0%). The same differences in the main characteristics between the psychiatric and non-psychiatric patients in A&E were observed in each phase (Table S1).

In Table 2, we report the main characteristic of the psychiatric patients admitted to A&E during the pre-pandemic period and after the spread of SARS-CoV-2.

Comparing the type of access to A&E, a difference was found only between phase 1 and phases 2 (*p* = 0.01) or 3 (*p* < 0.001). In phase 2 and phase 3, there was an increase in the number of patients who came to the A&E by ambulance after calling the emergency number. During the pandemic phase with the strongest restrictions (phase 2), we observed a significant increase in the number of patients with “psychomotor agitation” compared to the pre-pandemic phase 1 (72.5% vs. 62.3%,). In phase 3, there was an increase in hospitalizations after visits to the A&E and a decrease in home discharge compared to phases 1 and 2. No difference was found in the comparison of the percentage of visits to A&Es from municipalities with different classes of deprivation. However, during the first period of the pandemic, an increase in the access for psychiatric diseases was observed from people who live in areas with a lower DI. The incidence of access for psychiatric disease (IPd) from every municipality was calculated by the phases of the pandemic (Table S2), and a significant correlation was found only between the DI and IPd during phase 2 (r = 0.54, *p* = 0.003).

The rate of access for a psychiatric disorder for every thousand patients admitted to the A&E during the period under consideration was different. In the pre-pandemic period, the rate was 4.4; in the first year of the pandemic, the rate increased to 6.5; and in the second year, it was 4.6.

A generalized linear model using the Poisson distribution was applied to the incidence of entrance to an A&E for a psychiatric disease (IPd), using as covariates the age class, sex, DI class, phase (period of pandemic), and the interaction between the DI class and phase. All the covariates and interactions were statistically significant according to the generalized score test for type III contrast (for interaction, the *p*-value was 0.01; for all other variables, it was *p* < 0.001). The estimated IPd (cases per 1000 access ± standard error) for males was 5.23 ±0.23, and it was 2.86 ± 0.15 for females. The age classes with the highest estimated IPd were 21–40 (6.27 ± 0.3), followed by 41–60 (5.11 ± 0.26) and 13–20 (3.91 ± 0.39). Instead, people older than 60 years had a lower IPd (1.79 ± 0.12). In the period preceding the spread of SARS-CoV-2, the IPd was equal to 3.33 ± 0.19; after the pandemic began, there was an increase in the IPd to 4.74 ± 0.32 for phase 2 and 3.68 ± 0.25 for phase 3. The IPd for municipalities with the highest DI was the highest (7.26 ± 0.29), while those with medium, low, and very low DI were 3.02 ± 0.3, 3.21 ± 0.24, 3.18 ± 0.21, respectively.

**Figure 1:**
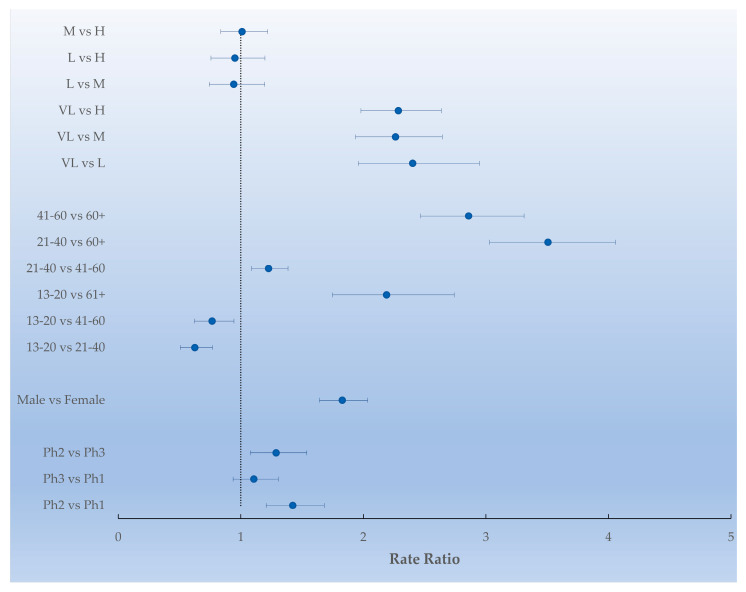
Forest plot of the rate ratios and their adjusted 95% CI between DI levels, sex, age group, and phases. (H, high DI; M, medium DI; L, low DI; VL, very low DI; Ph1, pre-pandemic period; Ph2, first year of the pandemic; Ph3, second year of the pandemic).

A significant difference for psychiatric access was found between the first year of the pandemic and both the pre-pandemic period and the second year of the pandemic (Rate Ratio 1.42 [IC 95% 1.20–1.68], RR 1.29 [IC 95% 1.08–1.54]), while no difference was found between the pre-pandemic period and the second year of the pandemic (RR 1.10 [IC 95% 0.9–1.30]). The RRs for sex and the age group were all significant, except the comparison between the age groups of 13–20 years and 41–60 years (*p* = 0.06). In general, young people had a greater risk of going to an A&E for a psychiatric disorder than older people. Only the comparison between the area with a very low DI and the others DI classes was significant (Figure 1).

**Figure 2:**
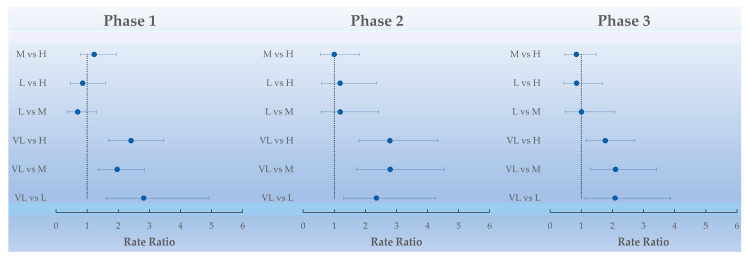
Forest plot of the rate ratios and their adjusted 95% CI between the DI levels for each phase. (Phase 1, pre-pandemic period; Phase 2, first year of the pandemic; Phase 3, second year of the pandemic; H, high DI; M, medium DI; L, low DI; VL, very low DI).

Is interesting to note that the interaction between the DI and the phases was significant only for the class with a very low DI compared to the other DI (low, medium and high). During phase 2 (first year of the pandemic), the RR decreased slightly for the very low DI versus the low but increased for the very low DI versus the medium and high (Figure 2).

## 4. Discussion

In our study we evaluated the short- and medium-term effects of SARS-CoV-2 infection and socioeconomic deprivation on psychiatric admissions to A&E departments. Overall, many studies have focused on the short-term effect of public health restrictions on admission to A&E [39] and on admissions for chronic and acute disease (e.g., coronary acute syndrome and malignancies) [6]. Other authors underlined the effect of the spread of infection on the mental health of people who did not have a psychiatric disease [40] and those who did [41]. Another Italian study underlined the possible impact of SARS-CoV-2 on the emergency and urgent psychiatric services in two hospitals in the city of Milan [42]. However, most researchers investigated only the short-term effect of the pandemic, comparing the years before the pandemic with the first period of the pandemic and lockdown measures.

Furthermore, several reports have systematically reviewed how the pandemic’s impact on mental health can be influenced by systemic social inequities such as age, ethnicity, sex, low income, unemployment, and sociocultural characteristics such as education [43,44].

To the best of our knowledge, this is the first report investigating the demographic, socioeconomic, and clinical differences in psychiatric A&E admissions between the pandemic-free and COVID-19 pandemic periods, using data from 2019 to 2021.

Comparing psychiatric patients with others admitted to A&E, the former were younger and male and came to the hospital principally by ambulance after calling the emergency number.

After the spread of COVID-19, only a few people came to the A&E for all pathologies principally due to the fear of contagion [45]. Especially during the first months, the diffusion of the alert message of the pandemic and the absence of specific COVID-19 therapies and vaccines induced people to avoid the places in which there was a higher risk of infection. In Italy [42,45,46] and in other countries around the world [47,48,49], a general reduction in admissions to all wards, both clinical and surgical, was observed. In our work, we observed a reduction in all admissions to A&E units of 28% in the first year of the pandemic, not much different from the reduction observed in Lombardia in the same period, at 32%. It is important to underline that Lombardia and Veneto were the two regions most affected in Italy by SARS-CoV-2 [50]. In general, patients were admitted to A&E only for an urgent acute illness that put the patient at risk of death [51]. Despite the general decrease in admissions to A&E, psychiatric patient admissions increased during the pandemic; in fact, patients often came in for drug abuse, attempted suicide, altered mental health conditions, and domestic violence [48]. In our study, and that of Stirparo et al. [52], there was an observed increase in the transport to A&E by ambulance during the pandemic compared to previous years; only a small number of patients entered the hospital on their own. Because the increase in ambulance transport concerned both psychiatric and non-psychiatric patients, this indicates that people came to the hospital particularly for critical conditions. This was confirmed by the increase in mortality in hospitals [42,52] (not only COVID-19-related [12]) and outside of hospitals [53].

During the spread of SARS-CoV-2, mental health was compromised in people with and without a previous psychiatric disorders [54]. In an international study [45], people declared that the public health measures adopted to contain the spread of the virus with a total lockdown caused a moderately stressful period. This period of stress was the basis for an increase in alcohol consumption [55], with a consequential exacerbation of previous psychopathology or as a trigger for it [56], especially in young people [49]. Since the start of the pandemic, there have been growing efforts to develop national and international guidance, policies, and resources to address mental health and psychosocial support needs [57,58].

In our study, we found a relation between the rate of admission to A&E for psychiatric disease and the socioeconomic deprivation of the municipalities where patients lived. Socioeconomic deprivation plays an important role in mental health and in psychiatry admissions [59], with a different degree of impact based on the patient’s psychiatric pathology [60]. In our study, independent of the pandemic, people who lived in municipalities with a very low deprivation index had a higher risk compared to others of being admitted to A&E, probably because these patients and their families had a greater awareness of psychopathologies and of any acute events requiring medical intervention.

The pandemic modified the effect of socioeconomic deprivation in the province of Taranto. During the first year, with the spread of SARS-CoV-2, especially in the area with high deprivation [26], a decrease in admissions to A&E from the municipality with very low deprivation and an increase in the admissions for the other levels of deprivation was observed. This most likely occurred because psychiatric patients and their families in less deprived areas were more aware of the risk of contagion if they went to the hospital. This evidence suggests that political decision-makers need to understand how psychopathological awareness is distributed throughout the population. Therefore, further studies could be performed to analyze the influence of social deprivation, in general, on disease awareness.

There were some limitations to our study that need to be acknowledged. The use of administrative data, which are dependent on the accuracy of disease coding, is one such limitation. As a result, the quality of the data could be influenced by the skills and expertise of the operators, and the resulting counts of cases and rates could be biased (either underestimated or overestimated).

## 5. Conclusions

Our study indicated a difference in A&E admission rates for psychiatric conditions between the pre-pandemic period and the first two years of the pandemic, as well as between the two years of the pandemic. A considerable increase in the admissions for psychiatric disease was only observed during the initial spread of SARS-CoV-2. Patients who lived in the most deprived municipalities generally came to the A&E less than those in the other areas, probably because the patients and their families had less awareness of their mental health. Robust and urgent public health policies to address these issues are needed to reduce the pandemic’s impact on these conditions of frailty.

## Figures and Tables

**Table 1:** Main characteristics according to the principal illness at the A&E visit. Comparison between psychiatric illness and non-psychiatric illness.

Parameter	Psych (*n* = 1438)	nPsych (*n* = 289,872)	*p*	Significant Pairwise Comparisons
**Gender**				
Male	894 (62.2)	141,056 (48.7)	<0.001	
Female	544 (37.8)	148,816 (51.3)		
**Age**	42 [33–56]	54 [35–73]	<0.001	
**Type of access**				
Own car (A)	467 (32.5)	189,625 (65.4)	<0.001	A vs. B *
Ambulance (B)	961 (66.8)	94,089 (32.5)		B vs. C *
Other (C)	10 (0.7)	6159 (2.1)		
**Nationality**				
Italy	1395 (97.3)	281,489 (97.4)	0.803	
Foreign	39 (2.7)	7551 (2.6)		
**Discharge**				
Recovery (A)	241 (16.8)	53,952 (18.6)	<0.001	A vs. D *
Refused hospitalization (B)	47 (3.3)	8597 (3.0)		B vs. D *
Home discharged (C)	884 (61.5)	204,462 (70.5)		C vs. D *
Other (D)	266 (18.5)	22,861 (7.9)		
**Class of Deprivation**				
H (<−0.596)	251 (17.5)	77,633 (26.8)	<0.001	H vs. VL *
M (−0.596 to −0.194)	213 (14.8)	63,067 (21.8)		M vs. VL *
L (−0.194 to 0.341)	104 (7.2)	33,206 (11.5)		L vs. VL *
VL (>0.341)	870 (60.5)	115,966 (40.0)		

Data are shown as *n* (%) or Median [IQR]. Psych: Psychiatric illness in A&E; nPsych: Non-Psychiatric illness in A&E. H, high DI; M, medium DI; L, low DI; VL, very low DI. * *p* < 0.001.

**Table 2:** Main characteristics of the psychiatric patients admitted to A&E. Comparison between the three phases.

Parameter	Phase 1 (*n* = 610)	Phase 2 (*n* = 443)	Phase 3 (*n* = 385)	*p*	Ph1 vs. Ph2	Ph1 vs. Ph3	Ph2 vs. Ph3
**Gender**							
Male	389 (63.8)	278 (62.8)	227 (59)	0.299	-	-	-
**Age**	42 [37–55]	43 [33–55]	43 [31–59]	0.953	-	-	-
**Type of access**							
Own car	235 (38.5)	133 (30)	99 (25.7)	<0.001	0.01	<0.001	0.327
Ambulance	370 (60.7)	309 (69.8)	282 (73.3)				
Other	5 (0.8)	1 (0.2)	4 (1.0)				
**Nationality**							
Italy	591 (97.2)	435 (98.2)	369 (96.3)	0.262	-	-	-
Foreign	17 (2.8)	8 (1.8)	14 (3.7)				
**Discharge**							
Recovery	93 (15.3)	66 (14.9)	82 (21.3)	<0.001	0.702	<0.001	<0.001
Refused hospitalization	22 (3.6)	9 (2)	16 (4.2)				
Home discharged	396 (64.9)	300 (67.7)	188 (48.8)				
Other	99 (16.2)	68 (15.4)	99 (25.7)				
**Class of Deprivation**							
H (<−0.596)	104 (17.1)	68 (15.4)	79 (20.5)	0.093	-	-	-
M (−0.596 to −0.194)	102 (16.7)	54 (12.2)	57 (14.8)				
L (−0.194 to 0.341)	38 (6.2)	34 (7.7)	32 (8.3)				
VL (>0.341)	366 (60)	287 (64.8)	217 (56.4)				
**Diagnosis**							
Psychiatric disorder	188 (30.8)	102 (23.0)	106 (27.5)	0.016	0.01	0.762	0.188
Self-harm	42 (6.9)	20 (4.5)	25 (6.5)				
Psychomotor agitation	380 (62.3)	321 (72.5)	254 (66.0)				

Data are shown as *n* (%) or Median [IQR]. Ph1, pre-pandemic period; Ph2, first year of the pandemic; Ph3, second year of the pandemic H, high DI; M, medium DI; L, low DI; VL, very low DI.

## Data Availability

No new data was created or analyzed in this study. Data sharing is not applicable to this article.

## Supplementary Materials

The following supporting information can be downloaded at: https://www.mdpi.com/article/10.3390/life13040943/s1, Table S1: Main characteristics of patients. Comparison between psychiatric access and non-psychiatric access in A&E in each pandemic phase; Table S2: Correlation between the municipalities incidence of access for a psychiatric disease (IPd) in A&E and deprivation index during the three phases.
