# Supplementary material for: The Impact of the COVID-19 Pandemic and Socioeconomic Deprivation on Admissions to the Emergency Department for Psychiatric Illness: An Observational Study in a Province of Southern Italy
Source: Life (Basel). 2023 Apr 3;13(4):943. doi: 10.3390/life13040943 (PMC10143488; doi:10.3390/life13040943)
Supplement: Supplementary file 1 [file life-13-00943-s001.zip › life-2313075-supplementary.pdf]

## Supplementary Material

**Table S1.** Main characteristics of patients. Comparison between psychiatric access and non-psychiatric access in A&E in each pandemic phase.

| Parameter | Phase 1 | | | Phase 2 | | | Phase 3 | | |
|---|---|---|---|---|---|---|---|---|---|
| | **Psich** (n = 610) | **nPsich** (n = 138273) | ***p*** | **Psich** (n = 443) | **nPsich** (n = 67959) | ***p*** | **Psich** (n = 385) | **nPsich** (n = 83640) | ***p*** |
| **Sex** | | | | | | | | | |
| Male | 389 (63.8) | 65510 (47.4) | <0.001 | 278 (62.8) | 33723 (49.6) | <0.001 | 227 (59) | 41823 (50) | <0.001 |
| **Age** | 42 [37–55] | 54 [34–72] | <0.001 | 43 [33–55] | 55 [36–73] | <0.001 | 43 [31–59] | 55 [36–73] | <0.001 |
| **Type of access** | | | | | | | | | |
| Own car | 235 (38.5) | 94991 (68.7) | | 133 (30) | 43045 (63.3) | | 99 (25.7) | 51589 (61.7) | |
| Ambulance | 370 (60.7) | 39836 (28.8) | <0.001 | 309 (69.8) | 23488 (34.6) | <0.001 | 282 (73.3) | 30765 (36.8) | <0.001 |
| Other | 5 (0.8) | 3446 (2.5) | | 1 (0.2) | 1426 (2.1) | | 4 (1.0) | 1286 (1.5) | |
| **Nationality** | | | | | | | | | |
| Italy | 591 (97.2) | 134388 (97.4) | 0.6994 | 435 (98.2) | 65941 (97.4) | 0.3695 | 369 (96.3) | 81160 (97.4) | 0.2022 |
| Foreign | 17 (2.8) | 3552 (2.6) | | 8 (1.8) | 1793 (2.7) | | 14 (3.7) | 2206 (2.7) | |
| **Discharge** | | | | | | | | | |
| Dead in A&E | 0 (0) | 158 (0.1) | | 0 (0) | 169 (0.3) | | 1 (0.3) | 238 (0.3) | |
| Recovery | 93 (15.3) | 23546 (17) | | 66 (14.9) | 13364 (19.7) | | 82 (21.3) | 17042 (20.4) | |
| Refused hospitaliz. | 22 (3.6) | 4142 (3) | <0.001 | 9 (2) | 2139 (3.2) | <0.001 | 16 (4.2) | 2316 (2.8) | <0.001 |
| Home discharged | 396 (64.9) | 100995 (73) | | 300 (67.7) | 47337 (69.7) | | 188 (48.8) | 56130 (67.1) | |
| Other | 99 (16.2) | 9432 (6.8) | | 68 (15.4) | 4950 (7.3) | | 98 (25.5) | 7914 (9.5) | |
| **Class of Deprivation** | | | | | | | | | |
| H (<-0.596) | 104 (17.1) | 37767 (27.3) | | 68 (15.4) | 18349 (27) | | 79 (20.5) | 21517 (40.2) | |
| M (−0.596 to −0.194) | 102 (16.7) | 30018 (21.7) | <0.001 | 54 (12.2) | 14579 (21.5) | <0.001 | 57 (14.8) | 18470 (12) | <0.001 |
| L (−0.194 to 0.341) | 38 (6.2) | 15613 (11.3) | | 34 (7.7) | 7533 (11.1) | | 32 (8.3) | 10060 (22.1) | |
| VL (>=0.341) | 366 (60) | 54875 (39.7) | | 287 (64.8) | 27498 (40.5) | | 217 (56.4) | 33593 (25.7) | |

Data are shown as n (%) or Median [IQR]; Psich: Psichiatric illness in A&E; nPsich: Non-Psichiatric illness in A&E; H, high DI; M, medium DI; L, low DI; VL, very low DI; phase 1: pre pandemic period, phase 2: pandemic period during 2020, phase 3: pandemic period during 2021.

**Table S2.** Correlation between the municipalities incidence of access for a psychiatric disease (IPd) in A&E and deprivation index during the three phases.

| Municipality | DI class | DI | Ipd | | |
|---|---|---|---|---|---|
| | | | Phase 1 | Phase 2 | Phase 3 |
| Maruggio | High | −1.50 | 0.7 | 0.0 | 0.0 |
| Torricella | | −1.37 | 3.3 | 1.6 | 1.5 |
| Fragagnano | | −1.20 | 1.8 | 0.0 | 0.0 |
| Leporano | | −1.19 | 0.0 | 0.0 | 0.0 |
| Avetrana | | −1.03 | 2.0 | 2.9 | 1.5 |
| Crispano | | −0.98 | 0.6 | 1.3 | 0.5 |
| Roccaforzata | | −0.98 | 0.0 | 0.0 | 0.0 |
| Monteiasi | | −0.90 | 0.0 | 0.0 | 0.0 |
| Manduria | | −0.80 | 2.3 | 2.3 | 2.4 |
| Carosino | | −0.76 | 1.0 | 0.0 | 0.0 |
| San Marzano Di S. Giuseppe | | −0.68 | 1.4 | 2.5 | 4.8 |
| Martina Franca | | −0.60 | 0.5 | 0.3 | 1.0 |
| Lizzano | | −0.59 | 1.2 | 0.0 | 0.7 |
| Sava | | −0.59 | 2.8 | 2.2 | 0.9 |
| San Giorgio Ionico | Medium | −0.58 | 3.0 | 1.5 | 1.7 |
| Castellaneta | | −0.57 | 1.0 | 1.5 | 0.9 |
| Montemesola | | −0.53 | 0.0 | 2.5 | 0.0 |
| Palagiano | | −0.51 | 0.7 | 1.0 | 0.3 |
| Faggiano | | −0.50 | 0.0 | 2.8 | 4.5 |
| Pulsano | | −0.42 | 0.5 | 0.9 | 2.2 |
| Mottola | | −0.30 | 0.4 | 1.4 | 0.0 |
| Grottaglie | | −0.23 | 1.6 | 1.7 | 1.8 |
| Laterza | | −0.19 | 0.6 | 2.1 | 0.5 |
| Ginosa | Low | −0.11 | 0.7 | 1.9 | 2.9 |
| Massafra | | −0.09 | 0.5 | 1.0 | 1.5 |
| Monteparano | | −0.02 | 2.2 | 7.3 | 0.0 |
| Palagianello | | 0.08 | 2.2 | 3.6 | 0.0 |
| Taranto | Very low | 0.34 | 2.5 | 2.4 | 2.0 |
| Statte | | 0.83 | 1.5 | 2.9 | 1.7 |

Idp, Incidence of psychiatric access; DI, deprivation index. Spearman Correlation (r) between IPd and DI. During phase 1 r = 0.07, $p$-value = 0.735; during phase 2 r = 0.54, $p$-value = 0.03; during phase 3 r = 0.26, $p$-value = 0.17.
